# Endogenous Proliferation after Spinal Cord Injury in Animal Models

**DOI:** 10.1155/2012/387513

**Published:** 2012-12-20

**Authors:** Ashley McDonough, Verónica Martínez-Cerdeño

**Affiliations:** ^1^Department of Pathology and Laboratory Medicine, UC Davis, School of Medicine, 4400 V Street, Sacramento, CA 95817, USA; ^2^Institute for Pediatric Regenerative Medicine, Shriners Hospitals for Children Northern California, 2425 Stockton Boulevard, Sacramento, CA 95817, USA; ^3^Biochemistry, Molecular, Cellular and Developmental Biology Graduate Group, UC Davis, One Shields Avenue, Davis, CA 95616, USA

## Abstract

Spinal cord injury (SCI) results in motor and sensory deficits, the severity of which depends on the level and extent of the injury. Animal models for SCI research include transection, contusion, and compression mouse models. In this paper we will discuss the endogenous stem cell response to SCI in animal models. All SCI animal models experience a similar peak of cell proliferation three days after injury; however, each specific type of injury promotes a specific and distinct stem cell response. For example, the transection model results in a strong and localized initial increase of proliferation, while in contusion and compression models, the initial level of proliferation is lower but encompasses the entire rostrocaudal extent of the spinal cord. All injury types result in an increased ependymal proliferation, but only in contusion and compression models is there a significant level of proliferation in the lateral regions of the spinal cord. Finally, the fate of newly generated cells varies from a mainly oligodendrocyte fate in contusion and compression to a mostly astrocyte fate in the transection model. Here we will discuss the potential of endogenous stem/progenitor cell manipulation as a therapeutic tool to treat SCI.

## 1. Introduction

In contrast to the former dogma that states that the adult mammalian central nervous system (CNS) is a tissue incapable of cell proliferation [1, 2], neuroscientists currently acknowledge the phenomena of postnatal mitosis in intact and injured CNS tissue, including in the spinal cord [1–10]. Research directed to modify the rate of proliferation and the fate of the newly generated cells in the spinal cord may have the capacity to restore function after injury. Researchers have followed different experimental strategies to modulate proliferation and cell differentiation in the spinal cord, including the manipulation of the levels of growth factors [11–18], proteins in the glial scar [19, 20], inflammation [21–23], and factors known to impair regeneration [24, 25]. Here we will discuss the response of endogenous stem/progenitor cells and the potential of manipulating these stem/progenitor cells as a therapeutic tool to treat spinal cord injury (SCI). 

## 2. Spinal Cord Injury

An estimated 265,000 people in the United States suffer some form of SCI [26]. Patients with SCI have a reduced life expectancy and experience a variable degree of impairment of movement, sensation and urinary and bowel function. Common health complications are pneumonia, urinary tract infections, and septicemia, all of which may result in recurrent hospitalizations. The degree of physical impairment, costs associated with care, and life expectancy are directly related to the level and extent of injury [27]. Lifetime costs for a single patient with SCI in the USA are between 1.1 and 4.3 millions of dollars [26]. Higher level and/or more complete injuries generally have a poorer prognosis and higher costs of care, while individuals with lower level and/or incomplete injuries typically have better clinical outcomes. 

SCI consists of a primary injury that leads to a secondary injury cascade. The primary injury is a physical insult, commonly induced by a compressive force of the vertebrae on the spinal cord [21]. This mechanical injury severs axons, causes necrotic cell death, and disrupts the vasculature. Consequently, primary injury leads to edema and ischemia, thus triggering a secondary injury cascade that consists of inflammation and the release of free radicals and cytotoxic levels of excitatory amino acids. This secondary injury cascade causes a further damage to axons and contributes to the death of numerous cell types [21, 39–41]. The terms primary and secondary injury should not be confused with acute and chronic SCI, which refers to the amount of time that has passed since the primary injury. Acute SCI is the first two weeks after injury, when secondary injury mechanisms are predominant and therapies for SCI are most effective, while the term chronic SCI refers to periods of time greater than six months after injury [21]. The majority of cell proliferation occurs during the acute phase of SCI [39]; thus this paper will focus on cell proliferation during the acute phase of SCI. 

Studies on cell response to SCI in humans are very limited due to issues of patient consent, technological constraints, medical urgency, and tissue availability. Consequently, animal models for SCI form the basis for much of our current knowledge on how cells in the spinal cord are affected by injury. The most common clinical presentation of SCI in human patients is a fracture dislocation injury in which the vertebrae are compressed against the spinal cord. The contusion model of animal SCI mimics this injury by applying a force onto the dorsal aspect of the spinal cord. By using this model on laboratory rodents, we can gain a better understanding of the precise events that take place in the spinal cord after injury and how regenerative therapies may modulate cellular responses to injury. 

Spinal cord injury causes rapid loss of all cell types within the spinal cord. In a model of incomplete contusion (10 g weight from a height of 2.5 cm), the rat spinal cord lost approximately 66% of the ventral motor neurons at the injury epicenter within 15 minutes of injury, and there was a near complete loss within four hours [42]. Oligodendrocytes expressing the marker antiadenomatosis polyposis coli clone CC-1 (CC1) and astrocytes expressing the marker glial fibrillary acidic protein (GFAP) also decreased within 15 minutes to 60% and 90% of uninjured spinal cord, respectively. While oligodendrocyte numbers did not recover during the acute injury phase, the number of astrocytes rebounded within 24 hours. Adjacent tissues also demonstrated the loss of glial and neuronal cells within 15 minutes of injury. Loss of all cell types is a prominent feature of SCI and occurs in all injury models, despite to varying degrees depending on injury type and severity [17, 21, 33, 35, 43, 44]. Stem/progenitor cells are also susceptible to injury. Horky et al. administered the proliferative marker bromodeoxyuridine (BrdU) prior to injury to label endogenous proliferating cells in the spinal cord. The number of BrdU^+^ cells labeled decreased by 67% within one day of a hemisection injury in mouse [30]. Approximately half of those proliferative cells expressed neuron-glial antigen 2 (Ng2), a marker of oligodendrocyte progenitor cells (OPCs). McTigue et al. also reported that approximately half of the endogenous OPCs were lost in the rat spinal cord after contusion injury, as well as a large number of oligodendrocytes within the injury epicenter [33], a result corroborated in a mouse model of contusion injury [17]. The loss of OPCs and oligodendrocytes may account for the delay in recovery of mature oligodendrocytes after injury. Axonal demyelination peaks during the first week, and is followed by remyelination that begins within 14 days of injury concomitantly with a recovery in the number of mature oligodendrocytes [33]. After injury, there is a tissue shrinkage in areas caudal to the lesion with more severe injuries resulting in a greater reduction in the spinal cord volume [1, 36]. To mimic SCI in human, researchers use a variety of animal models and injury types.

## 3. Models of Spinal Cord Injury

Valuable animal research models for SCI should demonstrate reproducible deficits in locomotor and sensory behaviors. Furthermore, SCI animal models should be adjustable to produce injuries of differing severity, which must correlate with the degree of neurological deficit observed. There are many forms of SCI, both clinically observed and experimentally validated, that can be classified into three groups of models based on the type of injury: transection, contusion, and compression.

**Figure 1:**
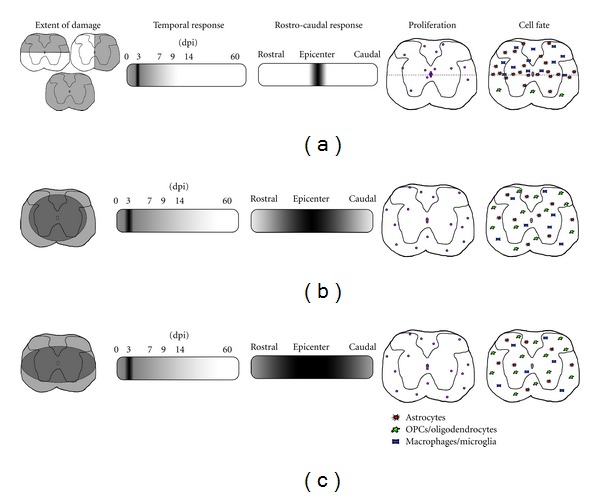
Qualitative summary of the features of the three types of animal models of SCI. Diagrams were created based on the references listed in Table 1. (a) Examples of transection injuries include dorsal hemisections (left), lateral hemisections (right), and complete transection (bottom). Proliferation peaks at 3 dpi and tapers off by 9 dpi. The mitotic response to injury is localized to the epicenter. Transection injuries are characterized by daughter cells acquiring astrocyte and macrophage/microglia fates. These cell types migrate to the region of injury. Depicted in the two right-most diagrams is proliferation and cell fate preferences in a dorsal hemisection injury. (b) A contusion injury typically results in a rim of spared white tissue. In response to injury, proliferation peaks at 3 dpi and is elevated for 14 days. Contusion injuries are characterized by a proliferative response that spans the rostrocaudal extent of the spinal cord. Cell fate of mitotic cells trends towards the oligodendrocyte lineage, but astrocytes and microglia also represent a portion of dividing cells. (c) Compression injuries closely resemble contusion injuries both in the extent of the spinal cord affected and cell types generated.

Transection SCI models are all forms of laceration to the spinal cord. Some common transection models include complete transection, in which a blade is passed through the entirety of the dura mater and spinal cord, dorsal or lateral hemisection, in which a blade is passed only half-way through the spinal cord (Figure 1(a)), and incision or minimal injury models, in which small nicks or cuts are made through the dura into the spinal cord. The pathological features of transection include a complete severance of axons in the area transected and the formation of a connective tissue mass and glial scar comprised of meningeal fibroblasts and astrocytes. Lacerations are rarely seen clinically, but may be present in instances of knife or gunshot wounds. Transection injuries are an attractive SCI model in experimental studies which aim to direct axonal growth through the glial scar [45]. 

Contusion injuries are produced by a focal force acting on the spinal cord, most commonly from the dorsal aspect [45]. Features of this experimental model include an intact glial limitans and pia mater and the formation of cavities within the spinal cord, a secondary complication known as syringomyelia in humans. Contusion injuries are characterized by a rim of spared white matter and cytoarchitectonic disorganization (Figure 1(b)) [1]. Experimentally, contusion injuries are performed using a weight drop method or dedicated apparatus such as the NYU or OSU impactor [46]. In contusion models, the severity of injury is controlled by increasing the weight or height from which the impactor tip is dropped. 

Compression injuries closely resemble contusion injuries in terms of pathological properties and neurological impairment; however, they differ in several regards. Contusion injuries are focal dorsal compressions, while in compression models the force can be applied either laterally or dorsally depending on the apparatus used. A compression injury can be generated using the Plemel method of calibrated forceps compression [47], aneurysm clips [45], or by applying a known weight directly onto the spinal cord [38]. The injury produced by compression generally affects a broader rostrocaudal area than contusion and depending on the application of force, may affect the lateral spinal cord more than the dorsal. Depending on the degree of severity, a compression injury may closely resemble a contusion injury in terms of pathology and cytoarchitectural distortions (Figure 1(c)) [47]. Injury severity is increased by compressing the spinal cord to a smaller diameter, by choosing aneurysm clips capable of applying greater pressure, or by increasing the amount of time the apparatus compresses the spinal cord. Depending on the method used and injury severity, the glial limitans or pia may be lacerated [45]. 

## 4. Proliferation in the Intact Spinal Cord

Neural stem cells (NSCs) are inherently difficult to identify and study. This is due in a large part to their heterogeneity of morphology and marker expression, as well as the lack of an identified anatomical niche [48–52]. The heterogeneity of marker expression in NSCs results in a significant overlap in marker expression between NSCs and mature astrocytes [48–57], which further complicates efforts to identify which cell types are NSCs and where they reside within the spinal cord. To be classified as a stem cell, a CNS cell should be able to self-renew and to give rise to all types of mature neural cells including neurons, astrocytes, and oligodendrocytes [2, 8, 28, 58, 59]. To test neural “stemness” researchers isolate potential stem cells and plate them in culture to form neurospheres that are further passaged to generate secondary neurospheres. Proliferative cells are considered neuronal stem cells if they form primary and secondary neurospheres and if cells differentiate into neurons, astrocytes, and oligodendrocytes *in vitro *[2, 8, 58, 60]. Fate restricted precursor cells capable of self-renewal, but limited to what fates their daughter cells can acquire, are not considered stem cells and are referred as progenitor cells [4, 60]. An example of progenitor cells in the spinal cord are OPCs, which are capable of self-renewal and produce oligodendrocytes. Intriguingly, neurospheres derived from the spinal cord exhibit multipotency *in vitro *and are capable of becoming neurons when transplanted into the hippocampus, but when transplanted into the spinal cord they only acquire glial fates [60]. Thus, some cells in the spinal cord are multipotent under certain conditions, but the native environment of the spinal cord is not conducive to fully realizing this potential. This disparity between *in vitro* capability and *in vivo* behavior raises the question of whether or not spinal cord cells capable of forming neurospheres *in vitro* are truly multipotent stem cells. In this paper we will discuss experiments used to identify populations of cells within specific regions of the spinal cord with capacity to self-renew and generate daughter cells that express markers of mature CNS cells. 

Although early studies showed postnatal neurogenesis in the brain [61–63], studies by Weiss et al. [2], Kehl et al. [7], Shihabuddin et al. [58], and Johansson et al. [8] were among the first to isolate and characterize stem/progenitor cells in the adult mammalian spinal cord. Weiss et al. isolated cells from the adult mouse spinal cord and, by culturing cells with epidermal growth factor (EGF) and basic fibroblast growth factor (FGF2), generated neurospheres capable of differentiating into all three major CNS cell types: neurons, oligodendrocytes, and astrocytes [2]. Shihabuddin et al. achieved similar results on tissue isolated from adult rat spinal cord, although they determined that FGF2 alone was sufficient for the isolation and culture of spinal cord stem/progenitor cells [58]. Similarly, Kehl et al. isolated and characterized stem/progenitor cells from the entire postnatal rat cervical spinal cord, but cultured these cells under monolayer conditions [7]. Under such conditions, cells were capable of mitosis and differentiation into functional neurons. These early reports were significant in that they described the presence of multipotent progenitors in the adult spinal cord. Neither of these studies described where the cells were located within the spinal cord, their lineage, or what markers they expressed *in vivo*. Later studies by Johansson et al. [8] and Yamamoto et al. [28] provided evidence of stem/progenitor cell presence within the central canal and lateral parenchyma of the spinal cord, respectively. 

The ependymal layer of the spinal cord has received much attention due to its role during embryonic development as a neuroprogenitor cell niche, its relation to the ependymal and subependymal layers of the brain, and its role in the reconnection and regeneration of the spinal cord of lower vertebrates. The central canal of the postnatal vertebrate spinal cord is congruent with the ventricular system of the brain and is similarly lined with a pseudostratified layer of ependymal cells [8, 64, 65]. However, unlike in the brain, there is no underlying subventricular zone (SVZ) in the spinal cord. Ependymal cells are ciliated cells with the soma located at the ventricular surface and a basal process in contact with the pia [66]. During development, proliferation occurs at the ventricular surface of the spinal cord, followed by subsequent migration of the daughter cells away from the central canal and their differentiation [4, 67]. In nonmammalian vertebrates, the ependymal cells in the postnatal spinal cord retain proliferative and neurogenic properties in both normal and injured spinal cord [66, 68, 69]. However, in mammals, the proliferative properties of postnatal ependymal cells in the spinal cord are more restricted and the frequency of proliferation is lower [3, 8, 59, 65, 70]. 

Ependymal cells in the mammalian spinal cord express markers associated with neural stem cells such as Nestin, GFAP, brain lipid binding protein (BLBP), Sox2, Vimentin, Musashi1, alpha-type platelet derived growth factor (PDGFR-*α*), Sox3, FoxJ1, and Notch1 receptor [8, 32, 52]. However, ependymal cells are highly heterogeneous in their marker expression, making the characterization of ependymal cell subtypes difficult. Several studies on the turtle spinal cord utilizing transmission electron microscopy (TEM), immunohistochemistry, and electrophysiology have characterized many subtypes of ependymal cells and surrounding cells in contact with the central canal [71–73]. Molecular evidence indicates that some ependymal cells in the postnatal spinal cord express the neuronal markers doublecortin, HuC/D, and/or polysialylated-neural cell adhesion molecule (PSA-NCAM). Just as the marker expression profile of ependymal cells is variable, so are their morphologies. Ependymal cells in the dorsal and ventral poles of the central canal resemble radial glial cells in the brain with long basal processes in contact with the pia mater and a short apical process in contact with the ventricular surface [65, 74], while the remainder of ependymal cells have cuboidal or tanycyte morphologies [65, 71]. Ependymal cells in turtle closely match the marker expression profile and morphology in mammals, indicating that some properties of ependymal cells are evolutionarily conserved among vertebrates despite the differential regenerative capabilities of these organisms in response to SCI. Understanding the precise differences between nonmammalian and mammalian spinal cord may enable therapies for SCI in humans to be capable of evoking the same level of plasticity observed in lower vertebrates. 

Recent studies also identified stem/progenitor cell proliferation in regions of the spinal cord away from the central canal. Horner et al. described proliferation in regions of the lateral parenchyma in intact adult rat spinal cord [4]. By using a single pulse of BrdU and collecting the spinal cord one hour later, they discovered that most of the BrdU^+^ cells were located in the outer circumference of the spinal cord, and concluded that these cells could not have originated from the ependymal cells of the central canal. These BrdU^+^ cells were predominately Ng2^+^, but also coexpressed markers for astrocytes, such as S100 calcium binding protein b (S100b), and for oligodendrocytes, such as antioligodendrocyte marker clone NS-1 (RIP), but did not express the neuronal markers class III *β*-tubulin (Tuj1) or neuronal nuclei (NeuN). A similar study by the Frisén Laboratory, using transgenic mice with an inducible Cre-LoxP system for lineage tracing, found that only ependymal cells were able to generate neurospheres. Proliferating cells of the Olig2^+^ lineage in the parenchyma did not but self-renewed and generated mature oligodendrocytes, ascribing to them a role as OPCs [3]. On the other hand, work by Yamamoto et al. on rat spinal cord supported a stem/progenitor cell niche along the outer circumference of the adult spinal cord based on the ability of dissociated and cultured lateral parenchyma to form neurospheres [28]. The Yamamoto study suggested that there is only one cell population in the lateral parenchyma capable of neurosphere formation and multipotent differentiation into the three main CNS cell types, but provided no evidence to where exactly in the parenchyma this cell population may be located nor what this cell population may be in terms of cell lineage or marker expression. The Frisén study ruled out Olig2^+^ cells as a candidate cell population to fulfill the role of stem/progenitor cells observed in the lateral parenchyma by Yamamoto et al. and Horner et al. The existence of a stem/progenitor cell niche in the lateral parenchyma is further supported by a recent study by Petit et al. that proposed, based on morphology and marker expression, that some nonependymal proliferating cells located in the subpial white matter of the mouse spinal cord are putative adult radial glia (RG) [52]. These cells expressed a combination of neonatal RG markers such as BLBP, Reelin, glutate aspartamate transporter (GLAST), and the adult neural stem cell markers GFAP and Sox2. During embryonic development the transient expression of BLBP parallels neurogenesis, and there is evidence from transgenic mouse studies that nearly all neurons in the mouse brain are derived from BLBP^+^ RG [53]. Additionally, BLBP^+^ cells with RG morphology are localized to the central gelatinosa surrounding the central canal in the adult turtle [72, 75], are implicated in the reconnection of the turtle spinal cord after transection [68, 76] and are directly involved in the regeneration of the axolotl spinal cord after amputation of the tail [77]. In mammalian models of injury, BLBP^+^ cells do not reconnect the spinal cord but undergo mitosis at an increased rate after injury [52]. Confounding the identification of stem cells in the CNS is the overlap in marker expression between neural stem cells and astrocytes, especially with regards to BLBP and GFAP. Neural stem cells in the adult brain are astroglial cells derived from RG cells that may fulfill the same role in the adult brain as RG cells in the developing brain [50, 51, 54–57, 78–82]. Taking this data into context, the BLBP^+^ cells of Petit et al. may be analogous to RG-derived astroglia in the adult brain. However, due to similarities in marker expression, morphology, and response to injury, further study is needed to differentiate this cell population from functional astrocytes. BLBP^+^ cells from the subpial white matter of the spinal cord could be isolated and cultured under conditions conducive to neurosphere formation to determine if they represent a pool of multipotent cells in the lateral parenchyma of the adult spinal cord. 

As the predominant dividing cell type in the intact postnatal spinal cord [4], Ng2 expressing cells have received a great deal of scientific interest. Ng2^+^ cells are capable of forming self-renewing neurospheres *in vitro *that are only able to produce oligodendrocytes [83], express a heterogeneous assortment of cell markers, and respond to injury with increased mitosis [35, 36, 84, 85]. Cells expressing Ng2 are found throughout the grey and white matter of the intact rodent spinal cord [4, 86], although bipolar or unipolar Ng2^+^ cells in the white matter are preferentially associated with radial elements of the spinal cord [4]. During development, Ng2^+^ cells are closely associated with RG cells [87]; thus the association of Ng2^+^ cells with radial elements in the spinal cord and the affiliation of putative adult RG cells described by Petit et al. with these same elements [52] is similar to the relationship between RG and Ng2^+^ cells during development. Ng2^+^ cells are generally considered to be OPCs; however, there is a large body of evidence suggesting that there are two distinct Ng2^+^ cell populations and that only one of them functions as an OPC [17, 18, 83, 85, 86, 88, 89]. These two types of Ng2^+^ cells differ in morphology; one class of Ng2^+^ cells is stellate shaped with highly branched processes, while the other class of Ng2^+^ cells has a flat, epithelioid soma and fewer processes [83, 86, 89]. These subsets of Ng2^+^ cells differ in their response to injury and growth factor treatment, electrophysiology, and in their potential for acquiring specific cell fates [17]. 

Of further note is the distribution of stem/progenitor cells throughout the spinal cord along the rostral-caudal axis. Horner et al. found more BrdU^+^ cells within the outer circumference in the cervical and lumbar spinal cord than in the thoracic spinal cord [4], while Petit et al. found an increasing number of BLBP^+^ putative RG cells at the pial edge in a cervical to lumbar gradient [52]. Similarly, Weiss et al. found that a greater number of neurospheres were generated from lumbar and sacral spinal cord than from rostral levels of spinal cord [2], but it is difficult to compare these studies because of the different experimental methods used. Olig2^+^ cells can be accounted for by BrdU labeling in the Horner study, which labeled all mitotic cells, but Olig2^+^ cells did not give rise to neurospheres and would not be represented in the Weiss study. If the cells described by Petit et al. are true stem cells capable of neurosphere formation, their population gradient might explain the increased numbers of neurospheres generated from lumbar spinal cord by Weiss et al. Using neurosphere formation as a criteria for defining a pool of stem/progenitor cells, these studies provide evidence that there is a cervical to lumbar gradient of stem/progenitor cells, and that specific levels of the spinal cord may have the capacity to generate a more robust and efficient response to injury. 

## 5. Proliferation after Injury

Lower vertebrates, such as eel [69], axolotl [77, 90], and zebrafish [91], are capable of replacing motor neurons and making a complete functional recovery after SCI. Turtles are capable of a significant, albeit incomplete, recovery of motor function [68]. However, there is little evidence for neurogenesis in mammals after injury and spontaneous functional improvement is modest at best. Despite the different functional outcomes, both nonmammalian and mammalian spinal cords initiate a mitotic response to injury. The mouse spinal cord is capable of forming neurospheres *in vitro *[2, 8]; however, when the lateral parenchyma from the injured spinal cord was cultured, neurospheres formed at a greater frequency grew faster and were more robust than neurospheres cultured from intact spinal cord tissue [3, 28]. Furthermore, when mice were administered BrdU prior to transection injury and the spinal cord tissue was dissociated and cultured, most of the neurospheres originated from BrdU^+^ cells had proliferated in response to injury [28]. Neurospheres derived under these conditions were capable of generating neurons *in vitro*, indicating that some cells that proliferate in response to SCI are multipotent under specific conditions; however, the lack of neurogenesis after injury *in vivo* indicates that the postnatal spinal cord environment is not conducive to the generation of new neurons.

### 5.1. Proliferation in Transection Models

Proliferation at the central canal plays an important role in the reconnection of the transected spinal cord of lower vertebrates such as the eel [69], turtle [68, 76], and lizard [92]. In the eel, the cross-sectional area of the central canal increased two- to eight-fold after a transection injury. The central canal reformed and grew into a regenerating gap, then expanded dorsally, re-creating the dorsal horns, which met and fused within 20 days of injury. Concomitant with the formation of a bridge and dorsal expansion, ependymal proliferation increased during the first 20 days and then decreased after the fusion of the regenerated dorsal horns. Ultimately, within 30 days the eel was capable of the swimming behavior indistinguishable from uninjured eels [69]. In the turtle, immediately after injury a connective bridge, consisting of GFAP^+^ and BLBP^+^ cells, was formed between the resected ends of the spinal cord [68]. By the end of the second week after injury, axons invaded the cellular bridge and within two to three months functional recovery was significant but incomplete, with permanent deficits in stepping and coordination. 

In contrast to the significant functional recovery in lower vertebrates, the spinal cord of higher order vertebrates such as rat and mouse has a far more limited potential for spontaneous recovery of motor and sensory function after injury. However, like lower vertebrates, mammals experience cell proliferation and there is evidence of repair mechanisms in the form of the remyelination of damaged axons. One of the earliest studies to characterize mammalian ependymal cells as putative progenitor cells examined the ependymal response to incision injury in rats. The *in vivo* proliferation rate of spinal cord ependymal cells was very low, but after an incision in the dorsal funiculus, the proliferation of the ependymal layer increased 50-fold [8]. Ependymal cells labeled with the fluorescent dye Dil proliferated and migrated over four weeks towards the lesion site, where they contributed to glial scar formation and expressed GFAP. Similarly, after a mild transection injury in rat, performed with a 30-gauge needle to create a minimal lesion adjacent to the central canal [29], ependymal proliferation increased one day after injury and peaked three days after injury, at which time point Dil^+^ cells were present within 70 *μ*m of the central canal [29]. Migration of labeled ependymal cells continued up to 140 *μ*m from the central canal towards the lesion at later time points. Additional experiments on the proliferative response to a more severe transection injury by Horky et al. described a significant increase in BrdU^+^ cells after a dorsal hemisection injury in mouse [30]. These cells were localized to the dorsal columns, neighboring grey matter at the lesion epicenter, and at the central canal. The greatest rate of proliferation occurred between three and nine days after injury. BrdU^+^ cells of the ependymal layer expressed either S100b or Nestin at early time points, which diminished over time. Within the spinal cord parenchyma, BrdU^+^/Ng2^+^ cells were abundant at all time points, while mature oligodendrocytes expressing CC1 were present one week after injury.

As already discussed, Yamamoto et al. was able to generate neurospheres from the lateral parenchyma of both intact and injured rat spinal cord [28]. In a follow-up study, they characterized the expression of neurogenic transcription factors after a transection injury. Only after injury did some ependymal cells express Pax6, although this expression was transient and limited to the first week after injury. Some cells in the lateral parenchyma also transiently expressed Pax6, and cells in the dorsal horn briefly expressed Pax7. Only some of these Pax6^+^ or Pax7^+^ cells coexpressed BrdU. Due to the timing of their emergence and distance from the ependymal layer, the authors concluded that Pax6^+^ cells in the lateral parenchyma could not have migrated from the central canal. It is possible that these cells were upregulating Pax6 in response to injury and/or becoming mitotic and producing daughter cells that expressed Pax6. Expression of Nkx2.2 increased throughout the medial and lateral parenchyma in a temporal pattern similar to that observed in the Pax6^+^ and Pax7^+^ cell populations. Pax6, Pax7, and Nkx2.2 are master regulators that confer a regional identity on neuroprogenitor cells during the embryonic development, with Pax6^+^ cells giving rise to ventral motoneurons and oligodendrocytes [93–97], Pax7^+^ cells giving rise to dorsal interneurons [95, 98, 99], and Nkx2.2^+^ cells giving rise to primarily oligodendrocytes [100] and a subset of ventral neurons [95]. Additionally, Pax6 is expressed in the adult SVZ by RG cells [78–82]. Pax6 and Pax7 are not expressed in postnatal mammalian spinal cord tissue; however, they are in cells located in the adult spinal cord of lower vertebrates. These cells represent a neurogenic niche capable of responding to injury by undergoing mitosis and generating a variety of cell fates that contribute to functional recovery [72, 73, 77, 90, 91]. These regulators of cell fate determination activate downstream neurogenic transcription factors such as Neurogenin2 (Ngn2), Mash1, Hb9, Isl-1/-2, Lbx3, and NeuroD1 [95, 98, 99]. While Yamamoto et al. observed the expression of some of these downstream transcription factors *in vitro* in a recapitulation of embryonic development, they did not observe similar gene expression *in vivo *after a transection injury [59]. Neurospheres cultured in the presence of an antagonist of Notch1, which is an inhibitor of Ngn2 and Mash1 and a negative regulator of neurogenesis, gave rise to more Tuj1^+^ cells than neurospheres cultured without a Notch1 inhibitor. This study raises the hypothesis that Notch1 is a potential factor that contributes to limiting neurogenesis in the postnatal spinal cord and identifies Notch1 as a potential therapeutic target for regenerative therapies targeting endogenous stem/progenitor cells in the spinal cord. 

Studies from the Frisén Laboratory have attempted to address the debate of which cell population(s) function as neural stem cells *in vivo *after injury. They performed a dorsal funiculus injury in transgenic mice in which Foxj1^+^ ependymal cells, Cx30^+^ astrocytes, or Olig2^+^ oligodendrocytes were labeled for cell fate mapping purposes. Ependymal lineage cells increased four- to five-fold at the injury site compared to adjacent regions, while cells of the glial lineages increased twofold in response to injury [3, 32]. Cell proliferation occurred during the first two weeks after injury and remained at the same level after four months. Cell migration also occurred after injury, with the cells of the astrocyte lineage forming the periphery of the scar and ependymal lineage cells composing the core of the scar tissue. There was little difference in the number of transgenically labeled cell populations between segments adjacent to the injury site and control tissue, indicating that the astrocytic and ependymal reaction to injury is restricted to the lesion epicenter [3, 32]. Similar to their results in intact spinal cord, the authors found that only cells of the ependymal lineage had the potential to give rise to different cell types. An interesting observation is that ependymal proliferation also occurred in response to a lateral incision of the spinal cord, indicating that the severance of the ependymal cell processes in the midline is not necessary for activation; however it may augment the ependymal proliferative response to injury [32]. This may explain why other reports indicate that there is ependymal proliferation, but at lower levels than reported in transection models of injury [8, 29]. 

Proliferation after injury has also been observed in nonhuman primates (macaque) with varying degrees of transection injuries. Yang et al. described how a more severe injury correlated with increased numbers of BrdU^+^ cells both within the cervical lesion as well as in unlesioned lumbar segments of the spinal cord [31]. In the macaque spinal cord, 81% of BrdU^+^ cells in the lesion were Iba1^+^ microglia seven weeks after injury. These Iba1^+^ cells could derive from peripheral macrophages that infiltrated the damaged spinal cord tissue and proliferated. The issue of nonnative cell proliferation accounting for BrdU^+^ cells has been raised by other researchers and addressed by Horky et al., who performed a hemisection injury on bone marrow chimeric mice. They traced the migration of GFP-labeled bone marrow stromal cells into the spinal cord after injury and determined that less than 10% of BrdU^+^ cells originated from proliferating peripheral blood cells [30]. It is thus unlikely that a significant portion of the BrdU^+^/Iba1^+^ cells observed in the primate spinal cord after transection injury derived from cell infiltrates and instead may be the result of endogenous proliferating microglia or macrophages. Some proliferative cells in the macaque spinal cord also expressed Olig2, although this proportion declined over time as mature oligodendrocyte and astrocyte markers increased [31]. There was no evidence of neuronal differentiation after injury in the primate spinal cord. These reports differ from transection studies on rats and mice in that injury to the primate spinal cord evoked a proliferative response in distal regions of the spinal cord and generated a larger proportion of BrdU^+^ microglial cells, features similar to reported results in animal models of contusive and compressive SCI. 

Sellers et al. focused on the response of Ng2^+^ cells to injury. Their experiments demonstrated changes in the post-injury extracellular environment of the spinal cord after hemisection injury and the effect that these cues had on Ng2^+^. To label proliferating Ng2^+^ cells, which are likely to be OPCs, they used an Ng2 promoter-specific retrovirus [18]. This retrovirus labels only proliferating cells and therefore is used for birth dating studies. After injury, labeled cells expressed markers for GFAP^+^ astrocytes, Iba1^+^ microglia, and pericytes. This study produced several interesting results, such as the generation of S100b^+^ phagocytic astrocytes from Ng2^+^ cells, which appeared within 24 hours at the lesion epicenter and appeared to be engaged in clearing myelin debris. However, a significant finding was that Ng2^+^ cells generated at different time points after injury acquired different cell fates. Cells labeled with retrovirus on the first day after injury acquired an astrocyte fate by the third day, while cells labeled on the seventh day became oligodendrocytes expressing myelin basic protein (MBP). These changes in cell fate determination were closely linked to changes in growth factor expression within the spinal cord after injury. 

Despite differences in experimental methods, transection injury in rat or mouse spinal cord evoked a localized proliferative response, primarily of the ependymal cells; but some Ng2^+^ OPCs and Ng2^+^ non-OPCs in the parenchyma also entered a mitotic program. Although stem/progenitor cells generate a variety of cell types, the majority of newly born daughter cells acquired astrocytic fates (Figure 1(a)). Ependymal cells are capable of migrating to the location of injury and giving rise to multiple lineages. Proliferation reached a peak at three days after injury and continued to be elevated for one to two weeks, although the duration of the proliferative response to transection injury is slightly unclear due to variations in experimental methods between reports.

### 5.2. Proliferation in Contusion Models

Early studies of contusion injuries in rabbit revealed an increased mitotic index in the ependymal layer associated with an increase in the diameter of the central canal at the lesion epicenter [9, 10]. A later study on contusion injury in rat, which examined the relationship between injury severity and the amount of fiber ingrowth, also described an expansion of the central canal and an increase in ependymal cell division at early time points after injury [1]. However, this expansion and increase in proliferation were only present in sections rostral and caudal of the injury and not within the epicenter of the lesion. This discrepancy between studies could be due to the varying severities of injury. Takahashi et al. compared moderate and severe contusion injuries in rat and noted that cell mitosis within the ependyma initially decreased in response to injury; however, it later resumed at an increased rate compared to control spinal cord [34]. A more severe injury depressed proliferation for a longer period of time; but once it resumed the rate was higher than in less severe injuries. They also showed that Nestin and GFAP were upregulated in response to injury regardless of whether or not the ependymal cells expressed PCNA. In more severe injuries the number of ependymal cells expressing these markers increased despite a lack of other proliferative markers. Unlike in transection injuries, which feature a localized mitotic response, cell division at the central canal increased significantly in the cervical and lumbar spinal cord. Furthermore, the recovery of limb function correlated with an increase in PCNA^+^ ependymal cells. 

Later studies in a contusion injury of moderate severity in mouse [36] and rat [84] showed that the response to contusive injury is conserved between mouse and rat. BrdU^+^ cells were observed in the white matter and the outer circumference of the spinal cord within the injury epicenter and the surrounding tissue, although the distribution of cell types differed among these areas. At the epicenter most BrdU^+^ cells coexpressed Ox42, a marker for microglia/macrophages in rat, known as CD11b in the mouse. In regions rostral and caudal to the injury site, the majority of mitotic cells were Ng2^+^ OPCs, followed in number by CC1^+^ oligodendrocytes, indicating some OPCs matured into oligodendrocytes. Proliferation peaked three days after injury at the epicenter of the injury [29, 30, 36, 84]. Interestingly, BrdU labeling was asymmetric with regards to proximity to injury, with a greater increase in the spinal segments rostral of the lesion than in caudal segments. This asymmetrical proliferation was limited to proximal regions; in sections greater than four mm rostral or caudal to the injury there were no asymmetrical differences in mitotic cell counts. This asymmetrical response, and the fate of daughter cells in different segments of the spinal cord after injury, was corroborated by McTigue et al. [33]. One week after injury, the greatest number of mitotic cells were located in the medial spinal cord and dorsal horns. Since ependymal cells can migrate 70 *μ*m in three days [29] and there is no described parenchymal stem cell niche, one week was insufficient to provide evidence for a stem/progenitor cell niche in either the parenchyma or in the ependymal layer. 

Yoo and Wrathall enriched Ng2^+^ cells isolated from injured rat spinal cord that were capable of self-renewal and formed small spheres; however, they gave rise to oligodendrocytes but rarely astrocytes [83], and thus, they characterized Ng2^+^ cells as fate restricted glial progenitors. Using a CNP-EGFP transgenic mouse, in which all cells expressing 2-3-cyclic nucleotide 3-phosphodiesterase (CNP) were labeled with enhanced green fluorescent protein (EGFP), Lytle et al. differentiated between Ng2^+^ OPCs and Ng2^+^ non-OPCs [17]. Both types of Ng2^+^ cells proliferated in response to injury; however, they exhibited distinct spatiotemporal population dynamics, with Ng2^+^ OPCs reaching a proliferative peak three days after injury and non-OPCs reaching a peak seven days after injury. Although both Ng2^+^ cell types upregulated Nestin in response to injury, non-OPCs did not express CC1 at any time. Furthermore, Ng2^+^ OPCs followed a developmental program of oligodendrogliosis. During the acute phase of injury, Olig2 was upregulated, while Olig1 and Nkx 2.2 were downregulated. At later time points, Olig1 and Nkx2.2 were upregulated, while Olig2 expression decreased; cells that underwent such changes in their transcriptional profile ultimately became CC1^+^ oligodendrocytes. These findings contrast with an earlier report by Yamamoto et al. where there was no expression of these transcription factors in a rat model of transection [59]. It is more likely that the difference between these two studies is due to injury type rather than the choice of animal model.

In contrast to the stem/progenitor cell response to transection injury, a contusion injury triggers a systemic mitotic response through the entire rostrocaudal extent of the spinal cord (Figure 1(b)). The cell fate of daughter cells also varies between transection and contusion injury, with most daughter cells in the injury epicenter expressing markers of inflammatory cells (i.e., microglia/macrophages); and most cells in rostrocaudal regions acquiring OPC/oligodendrocyte fate. In the contusion model, astrocytes are also produced by stem/progenitor cells, but not neurons. However, the proliferative response to injury is similar in that regardless of transection or contusion injury, there is a proliferative peak observed three days after injury. 

### 5.3. Proliferation in Compression Models

Compression and contusion injuries are similar in their pathology and have comparable cellular responses to injury. Wu et al. found that the Ng2^+^ cell response to compression injury resembled contusion injury [38]. 24 hours after compression injury there was a slight increase of Ng2^+^ cells in the subpial white matter and dorsal funicles in regions five mm rostral and caudal to the injury. One week after injury, there was an increase of Ng2 immunoreactivity in the subpial white matter along with a decrease in immunoreactivity in the dorsal funicles. Many bipolar Ng2^+^ cells with long processes extending from the subpial region of the dorsal spinal cord colocalized with 3CB-2, a marker for RG cells. These results support the hypothesis that there is a population of stem/progenitor cells in the subpial white matter of the adult spinal cord with features of RG cells [28, 52]. 

Proliferation at the central canal has also been observed in compression models of SCI, similar to that in contusive models. An analysis of ependymal proliferation and expression of Nestin after clip compression injury reported an increase in Ki-67^+^ cells 24 hours after injury [37]. The ependymal label index decreased after seven days and returned to control levels by 14 days. This may be due to the migration of the newly generated cells away from the central canal, as reported by other groups in other injury models [3, 29]. Most of the Ki-67^+^ cells were located in the ependymal layer adjacent to the lesion, with the number of labeled cells decreasing with distance from the lesion [37]. Although the labeling index increased, the number of ependymal cells lining the central canal remained at control values for at least seven days. An increase was first observed at 14 days when the ependyma appeared to have multiple layers of cells. Interestingly, the severity of the injury did not affect the labeling index or distance from the injury epicenter at which Ki67^+^ cells were present. Within six hours of injury there was a little change in Nestin expression; however, 24 hours after injury there was an increase in immunoreactivity. At later time points Nestin expression continued to increase and spread beyond the ependymal layer; however, not all Nestin^+^ cells were simultaneously Ki-67^+^, providing further evidence that some stem/progenitor cells may upregulate neuronal stem cell markers but not undergo mitosis in response to injury. In minimal injury [29] and transection [59] SCI models, some ependymal cells upregulated Nestin or other neural stem cell markers, but did not express proliferative markers. These cells may alternatively represent a population of premitotic stem/progenitor cells that upregulate stem cell markers prior to entering S-phase and mitosis, thus explaining the lack of BrdU incorporation and negative staining for other markers of active proliferation. Another hypothesis is that some stem/progenitor cells may remain dormant in the spinal cord and reexpress embryonic markers in response to injury, but fail to follow a program of neurogenesis due to an inhibitory environment [59]. Further experiments in contusion models of SCI are necessary to determine if the expression of stem cell markers without, or prior to mitosis is a widespread feature of SCI or is dependent on injury type and/or severity. 

Although the compression model of SCI is not as popular as the contusion and transection models, they share common features such as stem/progenitor cell proliferation peaking at three days after injury. Furthermore, the pathological features of the post injury spinal cord and cell fate of daughter cells closely resemble endogenous responses to contusive injury (Figure 1(c)). 

## 6. Therapeutic Strategies for Spinal Cord Injury

One of the most promising tools for functional recovery after SCI is the replacement of cells lost as a consequence of trauma. Cell replacement can be achieved by the transplantation of exogenous stem cells, such as cells derived from embryonic stem cells (ESCs) [101, 102], Schwann cells [103], and olfactory ensheathing cells [21, 39, 104–110]. Another promising treatment is the direct transplantation of stem cell-derived neuronal subtypes into the spinal cord. ESC-derived motor neurons engrafted and integrated into the developing chicken spinal cord and into noninjured rat spinal cord [111]. Transplantation of astrocytes improved locomotor recovery in transected rats by promoting axonal growth [43, 112], as did the transplantation of OPCs by remyelinating axonal sheaths [113]. Although there is evidence that these transplanted cells engraft and improve motor function after SCI, the exact mechanisms of recovery are not fully understood. The significant functional recovery observed in SCI animal models has led to human clinical trials of stem cell therapies. Currently there are several stem cell therapies in Phase I of clinical testing to treat various spinal disorders, such as amyotrophic lateral sclerosis (ALS). These include trials aimed at replacing lost and diseased motor neurons utilizing fetal neuroprogenitors [114], mesenchymal stem cells (MSCs) [115], and olfactory ensheathing cells [116]. Results published thus far indicate that the surgical procedure and transplantation of nonallogeneic cells into the human spinal cord are safe and well tolerated; however, patients are being enrolled for further testing to verify safety and determine efficacy. Such therapies could also be applied for the treatment of traumatic spinal injury. Recently, the biotechnology company StemCells, Inc. announced at the 51st Annual Scientific Meeting of the International Spinal Cord Society that they had transplanted human NSCs into three patients with complete thoracic SCI. Despite having no sensation below the injury level prior to surgery, two of the three patients regained some degree of sensitivity to touch, heat, and electrical stimuli. StemCells, Inc. is currently enrolling a larger cohort of patients to continue Phase I safety testing and validate their initial findings. 

Despite the encouraging results from stem cell transplantation, the use of certain stem cells can raise ethical concerns, cells may be difficult to obtain in sufficient quantities for the treatment of human patients, and there are concerns about tumorigenicity and immunogenicity. Thus, manipulating endogenous stem/progenitor cells is an attractive alternative regenerative therapy for SCI. Although an endogenous mitotic program is activated in response to injury, there is a persistent tissue shrinkage indicative of a massive loss of cells. The goal of manipulating endogenous stem/progenitor cells is to increase proliferation to recuperate lost neurons, oligodendrocytes, and astrocytes. Differentiating stem/progenitor cells into neurons is a priority, as the endogenous response appears to be biased toward replacing OPCs and astrocytes rather than generating new neurons. A potential approach for manipulating the endogenous response to injury is the administration of growth factors or cytokines to modulate the milieu in which endogenous cells are proliferating and differentiating. 

Brain-derived neurotrophic factor (BDNF), glial cell-derived neurotrophic factor (GDNF), nerve growth factor (NGF), ciliary neurotrophic factor (CTNF), vascular endothelial growth factor (VEGF), and many other growth factors have neuroprotective and neurotrophic properties that ameliorate neuronal death after injury [15, 21, 22, 27, 117] and have positive effects on OPC proliferation, remyelination of axonal sheaths [13], and axonal sprouting [15]. Other factors may be useful for SCI treatment, including TNF*α*, which is implicated in OPC proliferation [118], and BMP family members which induce neurogenesis during development and maintain the adult stem cell niche in the SVZ of the cortex [119]. How best to deliver these factors to the spinal cord is a current challenge. Possible means of delivery include intraventricular injection, viral mediated gene therapy, nonviral delivery vehicles, intrathecal delivery to the site of injury, and systemic administration. A few of these approaches have already been tested in animal models of SCI and other models of CNS injury. 

Early reports on the isolation and culture of multipotent spinal cord progenitors indicate that EGF and/or FGF2 is necessary for neurosphere formation and the multipotential differentiation capabilities of spinal stem/progenitor cells *in vitro* [2, 58]. However, the number of FGF2^+^ cells increased after contusive SCI, as did the expression of the gliogenic factors CNTF and glial growth factor (GGF2) [35]. When GGF2 and FGF2 were administered systemically, the number of CC1^+^ oligodendrocytes and Ng2^+^ non-OPCs increased, while the total number of Ng2^+^ OPCs was unaffected by growth factor treatment [17]. The latter observation was attributed to the ability of Ng2^+^ OPCs to maintain their population number despite enhanced mitosis and differentiation of their progeny. The failure of FGF2 to induce multipotency *in vivo* suggests that regenerative therapies may need to be more sophisticated and targeted to a number of biochemical pathways. For example, Notch1 is upregulated during the first week after injury [59]. Notch1 inhibits neurogenesis; so the inhibition of Notch1 may potentially increase the level of neurogenesis *in vivo*. *In vitro* data reveals that neurospheres originating from injured spinal cord and cultured in the presence of a Notch1 inhibitor generated more Tuj1^+^ cells than neurospheres cultured without this inhibitor. A successful combination therapy may need two or more components, such as FGF2 to increase stem/progenitor cell proliferation and Notch1 to encourage neuronal differentiation. 

Other attempts to manipulate the *in vivo* environment and induce changes in cell fate determination include the direct injection of BMP4 into a hemisected spinal cord [18]. Sellers et al. noticed that cells generated seven days after injury primarily gave rise to oligodendrocytes and correlated with an increase in the levels of BMP4. To mimic the seventh day post injury environment, they injected BMP4 into the spinal cord 24 hours after injury. As a result, the total number of GFAP^+^ cells and CC1^+^ cells decreased at the injury site. Reducing astrocytes may be beneficial by reducing the occurrence of allodynia and/or reducing the amount of neuroinhibitory molecules secreted by reactive astrocytes [120]; however, reducing the number of oligodendrocytes will negatively impact functional outcomes by reducing the number of myelinating-capable oligodendrocytes. When Noggin, a BMP4 antagonist, was injected at the same time point to manipulate the post injury environment, GFAP^+^ cells increased and CC1^+^ cells decreased. Thus, there may be conflicting cues in the spinal cord that affect the ability of stem/progenitor cells to generate specific cell types, and factors which are neurogenic during developmental processes may not have the same effect on adult stem/progenitor cells. Further evaluation of growth factor effects after SCI are needed to develop optimal therapies to promote neurogenesis *in vivo*. Basic research to determine how and why spinal stem/progenitor cells behave so differently *in vitro* and *in vivo* may allow for the design of regenerative therapies that increase cellular plasticity after injury. There is a wealth of *in vitro *data that we can draw on to develop new combinatorial therapies. For example, *in vitro* studies suggest that Olig2 and Mash1 enhance the differentiation of NSCs into mature neurons [121]. Other studies have highlighted the role of Pax6 on neuroprogenitor proliferation and differentiation in culture. The overexpression of Pax6 in NSC cultures resulted in an increased number of neurons [122] and on a greater axonal growth [123]. Although Pax6 and Pax7 are transiently expressed after injury [59], the timing and level of expression may not be sufficient to induce neurogenesis. Further studies on the effects of these transcription factors *in vivo* should be performed to determine if they are capable of improving sensory and motor function after trauma. 

Administration of growth factors, or lineage specific transcription factors, by a viral vector is another method for introducing therapeutic factors to the spinal cord. Adenoviral mediated overexpression of BDNF and Noggin in the adult striatum encouraged resident NPCs to differentiate into functional neurons [124], a therapy which should be tested in SCI. In a rat transection model, retroviral overexpression of Ngn2 and Mash1, in combination with the growth factors FGF2 and EGF, increased the number of oligodendrocytes generated from proliferating NPCs and also gave rise to new neurons [16]. Because of safety concerns, nonviral delivery vehicles may be a preferable alternative. Nonviral delivery vehicles include polymers, lipids, nanoparticles, and peptides [125]. For example, the peptide Tet1, which binds to the neuronal ganglioside GT1b, was able to successfully transduce, with a high degree of specificity, a large number of NSCs in the brain with a vector containing luciferase and *β*-galactosidase [126].

Administration of growth factors can also be accomplished by transplanting stem cells that have undergone genetic modifications to express growth factors in a specific region of the spinal cord or CNS. MSCs expressing BDNF preserved a retinal and optic nerve function when transplanted into a rat model of chronic ocular hypertension [127], and improved motor function in SCI rats [128]. However, it was hypothesized that these functional improvements were due to increased axonal sprouting and neuroprotective mechanisms, rather than to a modulation of proliferation and/or differentiation of endogenous cells. Nevertheless, engineered MSCs that overexpress Noggin, NGF, IGF-I, Shh, and/or other factors may increase the neurogenic capabilities of the post injury spinal cord and/or promote the mitosis of existing OPCs and NPCs. Bretzner et al. reported that the transplantation of olfactory ensheathing cells resulted in some functional recovery after compression SCI, as did the intrathecal administration of BDNF [11]; however, when both factors were administered together, motor function was impaired. Similarly, Davies et al. found that culturing astrocytes with BMP4 enhanced their engraftment and the functional recovery of SCI rats [112], however astrocytes cultured with CNTF prior to transplantation failed to integrate and resulted in mechanical allodynia and thermal hyperalgesia [129]. More work needs to be done to determine the optimal combination of factors and delivery vehicles to replace spinal cord cells lost to trauma or disease. 

A number of reports published in the last decade on regenerative therapies for SCI highlight the discrepancy between the *in vitro* and *in vivo* stem/progenitor cell response to growth factors and transcription factor overexpression, as well as the complexity of treatment design. The response to injury is complex; thus there is need for regenerative treatments targeting more than one factor to not only change the post injury environment, but also to modulate progenitor cell fate.

## 7. Conclusion

Some aspects of the mammalian response to SCI are common regardless of the injury type. However, other features, such as the location, extent, and rate of cell proliferation, vary depending on the type and severity of the injury (Table 1, Figure 1). In most injury models, proliferation begins within 24 hours of injury. Generally, transection injuries have higher rates of proliferation, particularly of the ependymal cells. The majority of injuries, whether performed in rats or mice and regardless of injury type, elicit a proliferative peak three days after injury. The proliferative response declines within one or two weeks and is close to control levels within three to four months of injury. This time course appears to be shorter in transection injuries. The rates of proliferation correlate closely with the spontaneous recovery of some motor function in mice and rats [30, 34, 36, 84], as well as with improvements in motor function in lower vertebrates [68, 69, 76]. Another common phenomenon after any type of SCI is the migration of newly generated cells towards the injury site. For example, in transection models, cells migrated towards the transection gap [3, 29, 32] and in contusion and compression models, cells migrated towards the dorsal or lateral aspect of the spinal cord where the injurious force had been applied [38, 130]. In transection injuries the proliferative response is local, while in contusion and compression injuries there is a response spanning a greater extent of the rostrocaudal axis (Figure 1). This broader response is likely due to contusion and compression injuries affecting a larger area of spinal tissue. Several studies using a contusion model have reported more proliferative cells rostral to the epicenter than caudal to it [33, 84]. This asymmetry may be the consequence of disrupted axonal conductance or an aberration in the serotonergic input in regions caudal of the primary injury [131].

The cell types that proliferate after injury vary between experimental injury models. However, this phenomenon may be attributed to experimental factors, such as a limited focus of each study, the selection of a transgenic approach, or the choice of immunohistochemical markers (see Table 1). Some studies only examined the ependymal cell response to injury or that of a very specific cell population, such as Ng2^+^ or Olig2^+^ cells. Nevertheless, taken together, the literature suggests that ependymal cells of the adult spinal cord are heterogeneous in the nature and that there are distinct stem/progenitor cell populations located within the parenchyma and ependymal layer that proliferate and generate specific spinal cord cells after injury. In addition, generated cells acquire different fates depending on injury type (Figure 1). After transection, the majority of proliferative cells express astrocyte markers (GFAP^+^ or S100b^+^ cells) and contribute to the formation of a fibrous glial scar. However, in contusion and compression models the majority of new cells outside of the lesion express markers for OPCs (Ng2^+^) and later for mature oligodendrocytes (CC1^+^). Compressive or contusive injuries result in ischemic and necrotic conditions that contribute to the demyelination of spinal tracts and cell death; so the replenishment of mature myelinating cells is necessary to prevent further degradation of axons [132]. Proliferation of OPCs is a mechanism by which the spinal cord can restore signal transductance through spared axons. Therefore, the replacement of the oligodendrocyte population in response to treatment is an important goal to achieve to develop successful regenerative therapies. Although there is evidence of axonal sprouting after injury [1] and potential increased neurogenesis after some forms of injury [130], there is no evidence that lost or damaged neurons in the mammalian spinal cord can be replaced without a therapeutic intervention. Therefore, increasing the number of neuronal lineage cells may be an additional goal for the achievement of successful therapies. 

Due to ethical issues, safety concerns, and technical obstacles in the use of human stem cells for treatment, the modulation of endogenous stem/progenitor cells in the spinal cord represents an attractive alternative to embryonic or induced pluripotent stem cell transplantation. Since the proliferation of endogenous stem/progenitor cells occurs during the first three days after injury, therapeutic approaches should target time points before the third day. Therapeutic intervention should also be considered at later time points to adjust the milieu in which the cells are differentiating and maturing. Furthermore, as there are clearly differences in the endogenous response between the different types of SCI, these therapies will need to be optimized for injury type and severity. For example, inhibitors directed against glial scar associated extracellular matrix components will be more effective in transection injuries, where a glial scar is a pathological feature which inhibits axonal sprouting. Increasing the rate of neurogenesis should be a goal for all injury types; however in contusive injuries it may be beneficial to supplement endogenous astrocyte generation, as there is evidence that specific astrocyte subtypes may aid in functional recovery after SCI [43, 129], although some subtypes may result in impaired function and/or allodynia [120, 129]. Disagreeing reports on the effects of astrocytes on functional improvement after SCI highlight the sensitivity of the spinal cord to changes in cell populations and reflect a need for careful studies on the contribution of glial-neuronal interactions to regeneration. Laceration injuries to the spinal cord may have a better functional outcome if oligodendrogenesis can be increased to promote the remyelination of axons damaged by transection injury or other secondary injury processes, as well as to myelinate newly generated neurons by therapeutic intervention. In the era of personalized medicine, understanding how endogenous stem/progenitor cells proliferate and differentiate in response to different forms of SCI will enable the development of effective regenerative therapies.

## Figures and Tables

**Table 1:** Summary of studies on the endogenous proliferative response to spinal cord injury.

SCI model	Animal model	Proliferation at		Extent of proliferation		Cell fate of daughter cells	Reference
		Central canal	Parenchyma	Epicenter	Rostral/ caudal		
Transection (incision)	Rat	+	Not stated	+	Not stated	Astrocytes	Johansson et al. [8]
Transection (complete)	Rat	+	+	+	Not stated	Macrophages, astrocytes, and OPCs	Yamamoto et al. [28]
Transection (minimal injury)	Rat	+	Not stated	+	−	Astrocytes, Nestin^+^ NSCs	Mothe and Tator [29]
Transection (hemisection)	Rat	+	+	−	+	OPCs, OLs, and astrocytes	Horky et al. [30]
Transection (hemisection)	Macaque	Not stated	+	+	+	Macrophages, OPCs, OLs, and astrocytes	Yang et al. [31]
Transection (incision)	Mouse	+	+	+	−	Astrocytes, OPCs	Barnabé-Heider et al. [3] Meletis et al. [32]
Contusion (weight drop)	Rabbit	+	Not stated	+	+	Not stated	Vaquero et al. [9] Vaquero et al. [10]
Contusion (NYU impactor)	Rat	+	Not stated	−	+	Not stated	Beattie et al. [1]
Contusion (OSU impactor)	Rat	Not stated	+	+	+	Macrophages, microglia, astrocytes, OPCs, and OLs	McTigue et al. [33]
Contusion (weight drop)	Rat	+	Not stated	+	+	Astrocytes	Takahashi et al. [34]
Contusion (weight drop)	Rat	Not stated	+	+	+	astrocytes, OLs, macrophages, and microglia	Zai et al. [35]
Contusion (weight drop)	Mouse	Not stated	+	+	+	OPCs, astrocytes, OLs, and macrophages	Lytle and Wrathall [36] Lytle et al. [17]
Compression (clip)	Rat	+	Not stated	+	−	Nestin^+^ cells	Namiki and Tator [37]
Compression (clip)	Rat	Not stated	+	+	+	OPCs	Wu et al. [38]

## References

[1] Beattie MS, Bresnahan JC, Komon J (1997). Endogenous repair after spinal cord contusion injuries in the rat. *Experimental Neurology*.

[2] Weiss S, Dunne C, Hewson J (1996). Multipotent CNS stem cells are present in the adult mammalian spinal cord and ventricular neuroaxis. *The Journal of Neuroscience*.

[3] Barnabé-Heider F, Göritz C, Sabelström H (2010). Origin of new glial cells in intact and injured adult spinal cord. *Cell Stem Cell*.

[4] Horner PJ, Power AE, Kempermann G (2000). Proliferation and differentiation of progenitor cells throughout the intact adult rat spinal cord. *The Journal of Neuroscience*.

[5] Bareyre FM (2008). Neuronal repair and replacement in spinal cord injury. *Journal of the Neurological Sciences*.

[6] Gross CG (2000). Neurogenesis in the adult brain: death of a dogma. *Nature Reviews*.

[7] Kehl LJ, Fairbanks CA, Laughlin TM, Wilcox GL (1997). Neurogenesis in postnatal rat spinal cord: a study in primary culture. *Science*.

[8] Johansson CB, Momma S, Clarke DL, Risling M, Lendahl U, Frisén J (1999). Identification of a neural stem cell in the adult mammalian central nervous system. *Cell*.

[9] Vaquero J, Ramiro MJ, Oya S, Cabezudo JM (1981). Ependymal reaction after experimental spinal cord injury. *Acta Neurochirurgica*.

[10] Vaquero J, Ramiro MJ, Oya S, Manuel Cabezudo J (1987). Ependymal cell proliferation after spinal cord injury. *Surgical Neurology*.

[11] Bretzner F, Liu J, Currie E, Roskams AJ, Tetzlaff W (2008). Undesired effects of a combinatorial treatment for spinal cord injury—transplantation of olfactory ensheathing cells and BDNF infusion to the red nucleus. *European Journal of Neuroscience*.

[12] Gomes WA, Mehler MF, Kessler JA (2003). Transgenic overexpression of BMP4 increases astroglial and decreases oligodendroglial lineage commitment. *Developmental Biology*.

[13] McTigue DM, Horner PJ, Stokes BT, Gage FH (1998). Neurotrophin-3 and brain-derived neurotrophic factor induce oligodendrocyte proliferation and myelination of regenerating axons in the contused adult rat spinal cord. *The Journal of Neuroscience*.

[14] Arvanian VL, Horner PJ, Gage FH, Mendell LM (2003). Chronic neurotrophin-3 strengthens synaptic connections to motoneurons in the neonatal rat. *The Journal of Neuroscience*.

[15] Rong W, Wang J, Liu X (2012). Naringin treatment improves functional recovery by increasing BDNF and VEGF expression, inhibiting neuronal apoptosis after spinal cord injury. *Neurochemical Research*.

[16] Ohori Y, Yamamoto SI, Nagao M (2006). Growth factor treatment and genetic manipulation stimulate neurogenesis and oligodendrogenesis by endogenous neural progenitors in the injured adult spinal cord. *The Journal of Neuroscience*.

[17] Lytle JM, Chittajallu R, Wrathall JR, Gallo V (2009). NG2 cell response in the CNP-EGFP mouse after contusive spinal cord injury. *Glia*.

[18] Sellers DL, Maris DO, Horner PJ (2009). Postinjury niches induce temporal shifts in progenitor fates to direct lesion repair after spinal cord injury. *The Journal of Neuroscience*.

[19] Barritt AW, Davies M, Marchand F (2006). Chondroitinase ABC promotes sprouting of intact and injured spinal systems after spinal cord injury. *The Journal of Neuroscience*.

[20] Bradbury EJ, Moon LDF, Popat RJ (2002). Chondroitinase ABC promotes functional recovery after spinal cord injury. *Nature*.

[21] Rowland JW, Hawryluk GW, Kwon B, Fehlings MG (2008). Current status of acute spinal cord injury pathophysiology and emerging therapies: promise on the horizon. *Neurosurgical Focus*.

[22] Ruff RL, McKerracher L, Selzer ME (2008). Repair and neurorehabilitation strategies for spinal cord injury. *Annals of the New York Academy of Sciences*.

[23] Tu J, Liao J, Stoodley MA, Cunningham AM (2010). Differentiation of endogenous progenitors in an animal model of post-traumatic syringomyelia. *Spine*.

[24] Freund P, Schmidlin E, Wannier T (2006). Nogo-A-specific antibody treatment enhances sprouting and functional recovery after cervical lesion in adult primates. *Nature Medicine*.

[25] Freund P, Schmidlin E, Wannier T (2009). Anti-Nogo-A antibody treatment promotes recovery of manual dexterity after unilateral cervical lesion in adult primates—re-examination and extension of behavioral data. *European Journal of Neuroscience*.

[26] (2011). *Spinal Cord Injury Facts and Figures at a Glance*.

[27] Hawryluk GW, Rowland J, Kwon BK, Fehlings MG (2008). Protection and repair of the injured spinal cord: a review of completed, ongoing, and planned clinical trials for acute spinal cord injury. *Neurosurgical Focus*.

[28] Yamamoto SI, Yamamoto N, Kitamura T, Nakamura K, Nakafuku M (2001). Proliferation of parenchymal neural progenitors in response to injury in the adult rat spinal cord. *Experimental Neurology*.

[29] Mothe AJ, Tator CH (2005). Proliferation, migration, and differentiation of endogenous ependymal region stem/progenitor cells following minimal spinal cord injury in the adult rat. *Neuroscience*.

[30] Horky LL, Galimi F, Gage FH, Horner PJ (2006). Fate of endogenous stem/progenitor cells following spinal cord injury. *Journal of Comparative Neurology*.

[31] Yang H, Lu P, McKay HM (2006). Endogenous neurogenesis replaces oligodendrocytes and astrocytes after primate spinal cord injury. *The Journal of Neuroscience*.

[32] Meletis K, Barnabé-Heider F, Carlén M (2008). Spinal cord injury reveals multilineage differentiation of ependymal cells. *PLoS Biology*.

[33] McTigue DM, Wei P, Stokes BT (2001). Proliferation of NG2-positive cells and altered oligodendrocyte numbers in the contused rat spinal cord. *The Journal of Neuroscience*.

[34] Takahashi M, Arai Y, Kurosawa H, Sueyoshi N, Shirai S (2003). Ependymal cell reactions in spinal cord segments after compression injury in adult rat. *Journal of Neuropathology and Experimental Neurology*.

[35] Zai LJ, Yoo S, Wrathall JR (2005). Increased growth factor expression and cell proliferation after contusive spinal cord injury. *Brain Research*.

[36] Lytle JM, Wrathall JR (2007). Glial cell loss, proliferation and replacement in the contused murine spinal cord. *European Journal of Neuroscience*.

[37] Namiki J, Tator CH (1999). Cell proliferation and nestin expression in the ependyma of the adult rat spinal cord after injury. *Journal of Neuropathology and Experimental Neurology*.

[38] Wu D, Shibuya S, Miyamoto O, Itano T, Yamamoto T (2005). Increase of NG2-positive cells associated with radial glia following traumatic spinal cord injury in adult rats. *Journal of Neurocytology*.

[39] Eftekharpour E, Karimi-Abdolrezaee S, Fehlings MG (2008). Current status of experimental cell replacement approaches to spinal cord injury. *Neurosurgical Focus*.

[40] Kwon BK, Stammers AMT, Belanger LM (2010). Cerebrospinal fluid inflammatory cytokines and biomarkers of injury severity in acute human spinal cord injury. *Journal of Neurotrauma*.

[41] Stammers AT, Liu J, Kwon BK (2012). Expression of inflammatory cytokines following acute spinal cord injury in a rodent model. *Journal of Neuroscience Research*.

[42] Grossman SD, Rosenberg LJ, Wrathall JR (2001). Temporal-spatial pattern of acute neuronal and glial loss after spinal cord contusion. *Experimental Neurology*.

[43] Davies SJA, Shih CH, Noble M, Mayer-Proschel M, Davies JE, Proschel C (2011). Transplantation of specific human astrocytes promotes functional recovery after spinal cord injury. *PLoS ONE*.

[44] Gensel JC, Tovar CA, Hamers FPT, Deibert RJ, Beattie MS, Bresnahan JC (2006). Behavioral and histological characterization of unilateral cervical spinal cord contusion injury in rats. *Journal of Neurotrauma*.

[45] Onifer SM, Rabchevsky AG, Scheff SW (2007). Rat models of traumatic spinal cord injury to assess motor recovery. *ILAR Journal*.

[46] Basso DM, Beattie MS, Bresnahan JC (1996). Graded histological and locomotor outcomes after spinal cord contusion using the NYU weight-drop device versus transection. *Experimental Neurology*.

[47] Plemel JR, Duncan G, Chen KWK (2008). A graded forceps crush spinal cord injury model in mice. *Journal of Neurotrauma*.

[48] Alvarez-Buylla A, Kohwi M, Nguyen TM, Merkle FT (2008). The heterogeneity of adult neural stem cells and the emerging complexity of their niche. *Cold Spring Harbor Symposia on Quantitative Biology*.

[49] Goritz C, Frisen J (2012). Neural stem cells and neurogenesis in the adult. *Cell Stem Cell*.

[50] Merkle FT, Alvarez-Buylla A (2006). Neural stem cells in mammalian development. *Current Opinion in Cell Biology*.

[51] Merkle FT, Tramontin AD, García-Verdugo JM, Alvarez-Buylla A (2004). Radial glia give rise to adult neural stem cells in the subventricular zone. *Proceedings of the National Academy of Sciences of the United States of America*.

[52] Petit A, Sanders AD, Kennedy TE (2011). Adult spinal cord radial glia display a unique progenitor phenotype. *PLoS ONE*.

[53] Anthony TE, Klein C, Fishell G, Heintz N (2004). Radial glia serve as neuronal progenitors in all regions of the central nervous system. *Neuron*.

[54] Doetsch F, Caille I, Lim DA, Garcia-Verdugo JM, Alvarez-Buylla A (1999). Subventricular zone astrocytes are neural stem cells in the adult mammalian brain. *Cell*.

[55] Garcia ADR, Doan NB, Imura T, Bush TG, Sofroniew MV (2004). GFAP-expressing progenitors are the principal source of constitutive neurogenesis in adult mouse forebrain. *Nature Neuroscience*.

[56] Ihrie RA, Alvarez-Buylla A (2008). Cells in the astroglial lineage are neural stem cells. *Cell and Tissue Research*.

[57] Imura T, Kornblum HI, Sofroniew MV (2003). The predominant neural stem cell isolated from postnatal and adult forebrain but not early embryonic forebrain expresses GFAP. *The Journal of Neuroscience*.

[58] Shihabuddin LS, Ray J, Gage FH (1997). FGF-2 is sufficient to isolate progenitors found in the adult mammalian spinal cord. *Experimental Neurology*.

[59] Yamamoto SI, Nagao M, Sugimori M (2001). Transcription factor expression and notch-dependent regulation of neural progenitors in the adult rat spinal cord. *The Journal of Neuroscience*.

[60] Shihabuddin LS, Horner PJ, Ray J, Gage FH (2000). Adult spinal cord stem cells generate neurons after transplantation in the adult dentate gyrus. *The Journal of Neuroscience*.

[61] Altman J, Bayer SA (1990). Migration and distribution of two populations of hippocampal granule cell precursors during the perinatal and postnatal periods. *Journal of Comparative Neurology*.

[62] Bayer SA, Yackel JW, Puri PS (1982). Neurons in the rat dentate gyrus granular layer substantially increase during juvenile and adult life. *Science*.

[63] Eriksson PS, Perfilieva E, Björk-Eriksson T (1998). Neurogenesis in the adult human hippocampus. *Nature Medicine*.

[64] Bruni JE (1998). Ependymal development, proliferation, and functions: a review. *Microscopy Research and Technique*.

[65] Bruni JE, Reddy K (1987). Ependyma of the central canal of the rat spinal cord: a light and transmission electron microscopic study. *Journal of Anatomy*.

[66] del Bigio MR (2010). Ependymal cells: biology and pathology. *Acta Neuropathologica*.

[67] Smart IH (1972). Proliferative characteristics of the ependymal layer during the early development of the spinal cord in the mouse. *Journal of Anatomy*.

[68] Rehermann MI, Marichal N, Russo RE, Trujillo-Cenóz O (2009). Neural reconnection in the transected spinal cord of the freshwater turtle *Trachemys dorbignyi*. *Journal of Comparative Neurology*.

[69] Dervan AG, Roberts BL (2003). Reaction of spinal cord central canal cells to cord transection and their contribution to cord regeneration. *Journal of Comparative Neurology*.

[70] Bruni JE, del Bigio MR, Clattenburg RE (1985). Ependyma: normal and pathological: a review of the literature. *Brain Research*.

[71] Marichal N, García G, Radmilovich M, Trujillo-Cenóz O, Russo RE (2009). Enigmatic central canal contacting cells: immature neurons in “standby mode”?. *The Journal of Neuroscience*.

[72] Russo RE, Fernández A, Reali C, Radmilovich M, Trujillo-Cenóz O (2004). Functional and molecular clues reveal precursor-like cells and immature neurones in the turtle spinal cord. *Journal of Physiology*.

[73] Russo RE, Reali C, Radmilovich M, Fernández A, Trujillo-Cenóz O (2008). Connexin 43 delimits functional domains of neurogenic precursors in the spinal cord. *The Journal of Neuroscience*.

[74] Noctor SC, Flint AC, Weissman TA, Wong WS, Clinton BK, Kriegstein AR (2002). Dividing precursor cells of the embryonic cortical ventricular zone have morphological and molecular characteristics of radial glia. *The Journal of Neuroscience*.

[75] Trujillo-Cenóz O, Fernández A, Radmilovich M, Reali C, Russo RE (2007). Cytological organization of the central gelatinosa in the turtle spinal corel. *Journal of Comparative Neurology*.

[76] Rehermann MI, Santiñaque FF, López-Carro B, Russo RE, Trujillo-Cenóz O (2011). Cell proliferation and cytoarchitectural remodeling during spinal cord reconnection in the fresh-water turtle *Trachemys dorbignyi*. *Cell and Tissue Research*.

[77] McHedlishvili L, Epperlein HH, Telzerow A, Tanaka EM (2007). A clonal analysis of neural progenitors during axolotl spinal cord regeneration reveals evidence for both spatially restricted and multipotent progenitors. *Development*.

[78] Ma DK, Bonaguidi MA, Ming GL, Song H (2009). Adult neural stem cells in the mammalian central nervous system. *Cell Research*.

[79] Martínez-Cerdeño V, Cunningham CL, Camacho J (2012). Comparative analysis of the subventricular zone in rat, ferret and macaque: evidence for an outer subventricular zone in rodents. *PLoS ONE*.

[80] Noctor SC, Martínez-Cerdeño V, Kriegstein AR (2008). Distinct behaviors of neural stem and progenitor cells underlie cortical neurogenesis. *Journal of Comparative Neurology*.

[81] Noctor SC, Martinez-Cerdeño V, Ivic L, Kriegstein AR (2004). Cortical neurons arise in symmetric and asymmetric division zones and migrate through specific phases. *Nature Neuroscience*.

[82] Noctor SC, Flint AC, Weissman TA, Dammerman RS, Kriegstein AR (2001). Neurons derived from radial glial cells establish radial units in neocortex. *Nature*.

[83] Yoo S, Wrathall JR (2007). Mixed primary culture and clonal analysis provide evidence that NG2 proteoglycan-expressing cells after spinal cord injury are glial progenitors. *Developmental Neurobiology*.

[84] Zai LJ, Wrathall JR (2005). Cell proliferation and replacement following contusive spinal cord injury. *Glia*.

[85] Lytle JM, Vicini S, Wrathall JR (2006). Phenotypic changes in NG2^+^ cells after spinal cord injury. *Journal of Neurotrauma*.

[86] Horner PJ, Thallmair M, Gage FH (2002). Defining the NG2-expressing cell of the adult CNS. *Journal of Neurocytology*.

[87] Diers-Fenger M, Kirchhoff F, Kettenmann H, Levine JM, Trotter J (2001). AN2/NG2 protein-expressing glial progenitor cells in the murine CNS: isolation, differentiation, and association with radial glia. *Glia*.

[88] Sellers DL, Horner PJ (2005). Instructive niches: environmental instructions that confound NG2 proteoglycan expression and the fate-restriction of CNS progenitors. *Journal of Anatomy*.

[89] Berry M, Hubbard P, Butt AM (2002). Cytology and lineage of NG2-positive glia. *Journal of Neurocytology*.

[90] Schnapp E, Kragl M, Rubin L, Tanaka EM (2005). Hedgehog signaling controls dorsoventral patterning, blastema cell proliferation and cartilage induction during axolotl tail regeneration. *Development*.

[91] Reimer MM, Kuscha V, Wyatt C (2009). Sonic hedgehog is a polarized signal for motor neuron regeneration in adult zebrafish. *The Journal of Neuroscience*.

[92] Egar M, Simpson SB, Singer M (1970). The growth and differentiation of the regenerating spinal cord of the lizard, *Anolis carolinensis*. *Journal of Morphology*.

[93] Ericson J, Rashbass P, Schedl A (1997). Pax6 controls progenitor cell identity and neuronal fate in response to graded Shh signaling. *Cell*.

[94] Genethliou N, Panayiotou E, Panayi H (2009). Spatially distinct functions of PAX6 and NKX2.2 during gliogenesis in the ventral spinal cord. *Biochemical and Biophysical Research Communications*.

[95] Sun T, Pringle NP, Hardy AP, Richardson WD, Smith HK (1998). Pax6 influences the time and site of origin of glial precursors in the ventral neural tube. *Molecular and Cellular Neurosciences*.

[96] Henley BM, McDermott KW (2010). The expression of neuroepithelial cell fate determinants in rat spinal cord development. *Journal of Molecular Neuroscience*.

[97] Bel-Vialar S, Medevielle F, Pituello F (2007). The on/off of Pax6 controls the tempo of neuronal differentiation in the developing spinal cord. *Developmental Biology*.

[98] Alaynick WA, Jessell TM, Pfaff SL (2011). Snapshot: spinal cord development. *Cell*.

[99] Tanabe Y, Jessell TM (1996). Diversity and pattern in the developing spinal cord. *Science*.

[100] Tochitani S, Hayashizaki Y (2008). Nkx2.2 antisense RNA overexpression enhanced oligodendrocytic differentiation. *Biochemical and Biophysical Research Communications*.

[101] Cui Y-F, Xu JC, Hargus G, Jakovcevski I, Schachner M, Bernreuther C (2011). Embryonic stem cell-derived L1 overexpressing neural aggregates enhance recovery after spinal cord injury in mice. *PLoS ONE*.

[102] Krencik R, Weick JP, Liu Y, Zhang ZJ, Zhang SC (2011). Specification of transplantable astroglial subtypes from human pluripotent stem cells. *Nature Biotechnology*.

[103] Menei P, Montero-Menei C, Whittemore SR, Bunge RP, Bartlett Bunge M (1998). Schwann cells genetically modified to secrete human BDNF promote enhanced axonal regrowth across transected adult rat spinal cord. *European Journal of Neuroscience*.

[104] Obermair FJ, Schröter A, Thallmair M (2008). Endogenous neural progenitor cells as therapeutic target after spinal cord injury. *Physiology*.

[105] Nakamura M, Tsuji O, Bregman BS, Toyama Y, Okano H (2011). Mimicking the neurotrophic factor profile of embryonic spinal cord controls the differentiation potential of spinal progenitors into neuronal cells. *PLoS ONE*.

[106] Giménez Y Ribotta M, Orsal D, Feraboli-Lohnherr D, Privat A (1998). Recovery of locomotion following transplantation of monoaminergic neurons in the spinal cord of paraplegic rats. *Annals of the New York Academy of Sciences*.

[107] Lepore AC, Fischer I (2005). Lineage-restricted neural precursors survive, migrate, and differentiate following transplantation into the injured adult spinal cord. *Experimental Neurology*.

[108] Bonner JF, Connors TM, Silverman WF, Kowalski DP, Lemay MA, Fischer I (2011). Grafted neural progenitors integrate and restore synaptic connectivity across the injured spinal cord. *The Journal of Neuroscience*.

[109] Cummings BJ, Uchida N, Tamaki SJ (2005). Human neural stem cells differentiate and promote locomotor recovery in spinal cord-injured mice. *Proceedings of the National Academy of Sciences of the United States of America*.

[110] Feraboli-Lohnherr D, Orsal D, Yakovleff A, Giménez Y Ribotta M, Privat A (1997). Recovery of locomotor activity in the adult chronic spinal rat after sublesional transplantation of embryonic nervous cells: specific role of serotonergic neurons. *Experimental Brain Research*.

[111] Lee H, Al Shamy G, Elkabetz Y (2007). Directed differentiation and transplantation of human embryonic stem cell-derived motoneurons. *Stem Cells*.

[112] Davies JE, Huang C, Proschel C, Noble M, Mayer-Proschel M, Davies SJA (2006). Astrocytes derived from glial-restricted precursors promote spinal cord repair. *Journal of Biology*.

[113] Faulkner J, Keirstead HS (2005). Human embryonic stem cell-derived oligodendrocyte progenitors for the treatment of spinal cord injury. *Transplant Immunology*.

[114] Glass JD, Boulis NM, Johe K (2012). Lumbar intraspinal injection of neural stem cells in patients with amyotrophic lateral sclerosis: results of a phase I trial in 12 patients. *Stem Cells*.

[115] Mazzini L, Mareschi K, Ferrero I (2012). Mesenchymal stromal cell transplantation in amyotrophic lateral sclerosis: a long-term safety study. *Cytotherapy*.

[116] Chen L, Chen D, Xi H (2012). Olfactory ensheathing cell neurorestorotherapy for amyotrophic lateral sclerosis patients: benefits from multiple transplantations. *Cell Transplantation*.

[117] McCall J, Weidner N, Blesch A (2012). Neurotrophic factors in combinatorial approaches for spinal cord regeneration. *Cell and Tissue Research*.

[118] Arnett HA, Mason J, Marino M, Suzuki K, Matsushima GK, Ting JPY (2001). TNF*α* promotes proliferation of oligodendrocyte progenitors and remyelination. *Nature Neuroscience*.

[119] Bond AM, Bhalala OG, Kessler JA (2012). The dynamic role of bone morphogenetic proteins in neural stem cell fate and maturation. *Developmental Neurobiology*.

[120] Hofstetter CP, Holmström NAV, Lilja JA (2005). Allodynia limits the usefulness of intraspinal neural stem cell grafts; directed differentiation improves outcome. *Nature Neuroscience*.

[121] Uchida Y, Nakano SI, Gomi F, Takahashi H (2007). Differential regulation of basic helix-loop-helix factors Mash1 and Olig2 by *β*-amyloid accelerates both differentiation and death of cultured neural stem/progenitor cells. *Journal of Biological Chemistry*.

[122] Kallur T, Gisler R, Lindvall O, Kokaia Z (2008). Pax6 promotes neurogenesis in human neural stem cells. *Molecular and Cellular Neuroscience*.

[123] Sebastián-Serrano A, Sandonis A, Cardozo M, Rodríguez-Tornos FM, Bovolenta P, Nieto M (2012). Palphax6 expression in postmitotic neurons mediates the growth of axons in response to SFRP1. *PLoS ONE*.

[124] Chmielnicki E, Benraiss A, Economides AN, Goldman SA (2004). Adenovirally expressed noggin and brian-derived neurotrophic factor cooperate to induce new medium spiny neurons from resident progenitor cells in the adult striatal ventricular zone. *The Journal of Neuroscience*.

[125] Bergen JM, Park IK, Horner PJ, Pun SH (2008). Nonviral approaches for neuronal delivery of nucleic acids. *Pharmaceutical Research*.

[126] Kwon EJ, Lasiene J, Jacobson BE, Park IK, Horner PJ, Pun SH (2010). Targeted nonviral delivery vehicles to neural progenitor cells in the mouse subventricular zone. *Biomaterials*.

[127] Harper MM, Grozdanic SD, Blits B (2011). Transplantation of BDNF-secreting mesenchymal stem cells provides neuroprotection in chronically hypertensive rat eyes. *Investigative Ophthalmology and Visual Science*.

[128] Sasaki M, Radtke C, Tan AM (2009). BDNF-hypersecreting human mesenchymal stem cells promote functional recovery, axonal sprouting, and protection of corticospinal neurons after spinal cord injury. *The Journal of Neuroscience*.

[129] Davies JE, Pröschel C, Zhang N, Noble M, Mayer-Pröschel M, Davies SJA (2008). Transplanted astrocytes derived from BMP- or CNTF-treated glial-restricted precursors have opposite effects on recovery and allodynia after spinal cord injury. *Journal of Biology*.

[130] Ke Y, Chi L, Xu R, Luo C, Gozal D, Liu R (2006). Early response of endogenous adult neural progenitor cells to acute spinal cord injury in mice. *Stem Cells*.

[131] Murray KC, Nakae A, Stephens MJ (2010). Recovery of motoneuron and locomotor function after spinal cord injury depends on constitutive activity in 5-HT_2C_ receptors. *Nature Medicine*.

[132] Keirstead HS, Levine JM, Blakemore WF (1998). Response of the oligodendrocyte progenitor cell population (defined by NG2 labelling) to demyelination of the adult spinal cord. *Glia*.

